# Account of a Case of Prolapse of the Gravid Uterus during Labour

**Published:** 1848-04

**Authors:** E. F. Watson

**Affiliations:** North Carolina, Orange Co.


### *Account of a case of prolapse of the gravid Uterus during labour.* By E. F. Watson, M. D. *In a letter to the Editor.*

Dear Sir:—I have been induced to send you the following history of a case that occurred in my practice, and if you think it worthy you may give it an insertion in your valuable journal. Perhaps it may be interesting to some of your numerous readers who may happen to meet with one of a similar character, it being the first of the kind I have ever met with in a tolerably extensive practice during the last thirteen years.

On Thursday, the 13th of January last, about 8 o'clock in the morning, I was called in haste to visit Mrs. C——, aged about 22 years, in labour with her first child. On arriving at the house I found her in charge of two midwives, who had been with her the greater part of the preceding night. The patient I found lying on the floor, with a few bedclothes under and over her. I was requested to examine the case and do something immediately for the relief of the patient, as the midwives and friends were very much alarmed at her condition. Upon examination I found the uterus entirely without the vulva, containing a fœtus at full term of utero-gestation; when placing my hand on the uterus, I found it dry and ecchymosed in several places on the body and fundus, with the os uteri dilated to about the size of a twenty-five cent piece, and very thick, firm and unyielding, with the scalp slightly projecting through the orifice. Upon enquiry, I learned that early in the night the patient had been taken in labour, and the midwife first called to the case finding something unusual exhibiting itself, desired a physician to be sent for; the friends objecting, they sent for the second midwife, who thought herself able when she arrived to surmount every difficulty, and bring the labour to a speedy and successful termination. But after exhausting all her skill, and making the case no doubt a great deal worse by her efforts for delivery, she then advised the friends to send for a physician with all possible speed. I had the patient taken up and placed in bed as soon as possible, and applied linen clothes, rung out of warm water, to the uterus, and sent for Dr. J. S. Bracken to come to my assistance and bring the necessary instruments for delivery, having none with me but a common case of midwifery instruments and a small scalpel or two. After several applications of the linen (for I had them renewed every few minutes) I found the uterus began to act, and with such force as to give strong fears that a laceration of the organ might take place. The pains, though frequent and very strong, had no effect in enlarging the os uteri. Deeming the case too urgent to wait any longer for the arrival of Dr. Bracken, I determined to divide the neck of the uterus longitudinally, and for that purpose introduced my finger in the absence of pain, and with a common scalpel, my finger serving as a director and also protecting the scalp of the child, divided the neck to the extent of three-quarters of an inch or more. Another pain soon came on, which caused but little dilatation. Owing to the cartilaginous condition of the cervix, I thought it best to make two other incisions, about equidistant from the first, and to about the same extent; after which, the pains having returned, the fœtus was expelled in a state of asphyxia, but by repeated inflation of the lungs and using the common restoratives, it was soon discovered to breathe, and in a short time cried out lustily, to the gratification of mother and friends. The placenta was soon delivered without difficulty or hemorrhage, the uterus contracting firmly, as though it had been in its proper position. By this time Dr. Bracken having arrived, and having examined the case—the parts being cleansed—I then returned the uterus to its proper position, applied a large piece of sponge, well oiled, to the vagina, and secured the same by the application of a T bandage.

The patient appeared quite comfortable after delivery, with a pulse about 75, and had borne her sufferings with uncommon fortitude, having been in the condition described above about twelve hours. We left the patient about 12 o'clock, and ordered half an ounce of ol. ricini to be taken at 4 o'clock in the evening, and elm mucilage to be thrown up the vagina every six or eight hours, with a promise that I would see her the next day. Diet to be rice water or weak panada.

*Friday, 14th.* Visited the patient about 10 o'clock; found her quite comfortable, except slight tenderness of abdomen; pulse about 100; bowels had been twice moved during the night; after-pains but slight. Ordered oil to be repeated in the evening; fomentations to the abdomen, and to continue the injections per vaginam; left a weak solution of creosote to be used as a lotion.

*Saturday, 15th.* Dr. Bracken kindly visited the patient to-day, and found her greatly improved; tenderness of abdomen much relieved. Treatment to be the same, with oil to be taken next day. Diet the same.

*Monday, 17th.* Visited the patient and found her gradually improving; tenderness of abdomen entirely subsided; a free secretion of milk has taken place since my last visit. On removing the bandage and sponge, I discovered the uterus had come down so that its neck could be seen. The incisions seemed to be perfectly healed and the appearance of the parts quite healthy. Patient complains of scarcely any pain or tenderness about the vagina or os uteri in replacing it to its proper position. Ordered her diet to be a little more nourishing, and to continue the injections per vaginam twice a day, and creosote wash as heretofore.

*Wednesday, 19th.* Patient improving, and thinks herself able to sit up if she was permitted to do so. Bowels ordered to be kept open with oil, and to continue diet and treatment as directed at last visit, with strict direction to keep herself in the horizontal posture until next visit.

*Monday, 24th.* Visited patient and found her, as she expressed herself, entirely well, if she could only get permission to sit up, which I granted her, and directed her diet to be more nourishing, and to continue wearing the bandage and sponge, which would serve to support the parts in their relaxed condition; since which time I have had no further trouble with the patient, but she has continued to improve, and was soon able to attend to her ordinary occupation, and has suffered during her confinement but little more than if her case had been one of an ordinary character. The patient living at the distance of near ten miles from me, I have not seen her since my visit on the 24th, but have frequently heard from her, and have been gratified to hear that both mother and child are doing well.

Prof. Meigs, in speaking of deformity of the pelvis, says: "When labour comes on in a woman with a very ample pelvis, her throes have the effect of urging the whole body of the uterus, with all its contents, down towards the perineal outlet; and hence, before the mouth of the womb is fully dilated, the head of the child, still partially enveloped in the undilated cervix, may be pushed through the vulva."* In making a partial examination of the pelvis in the above case, I found it not larger than ordinary; but the cervix being completely cartilaginous, every pain did no more than force the uterus with its contents through the vulva; and it is entirely probable that the efforts made at delivery before I saw the patient, assisted materially in making the prolapsus what I found it on my arrival.

*North Carolina, Orange Co.*, March 3d, 1848.

---

To the Editor of the Medical Examiner.

Dear Sir:—At a Stated Meeting of the Norfolk Medical Society, held on the 4th of March, 1848, Thomas F. Andrews, M. D., and Richard W. Silvester, M. D., were elected Delegates to the National Medical Association to assemble in Baltimore in May. William J. Moore, M. D., and George L. Upshur, M. D., were appointed alternates.

Yours, &c.,

Geo. L. Upshur, M. D., Secretary.

*Norfolk*, (*Va.*,) March 13th, 1848.

* Practice of Midwifery, p. 29, Second Edition.

